# Identification of Factors Associated With Variation in US County-Level Obesity Prevalence Rates Using Epidemiologic vs Machine Learning Models

**DOI:** 10.1001/jamanetworkopen.2019.2884

**Published:** 2019-04-26

**Authors:** David Scheinker, Areli Valencia, Fatima Rodriguez

**Affiliations:** 1 Department of Management Science and Engineering, Stanford University School of Engineering, Stanford, California; 2 Department of Preoperative Services, Lucile Packard Children’s Hospital Stanford, Stanford, California; 3 Medical Student, Stanford University School of Medicine, Stanford, California; 4 Division of Cardiovascular Medicine, Stanford University School of Medicine, Stanford, California

## Abstract

**Importance:**

Obesity is a leading cause of high health care expenditures, disability, and premature mortality. Previous studies have documented geographic disparities in obesity prevalence.

**Objective:**

To identify county-level factors associated with obesity using traditional epidemiologic and machine learning methods.

**Design, Setting, and Participants:**

Cross-sectional study using linear regression models and machine learning models to evaluate the associations between county-level obesity and county-level demographic, socioeconomic, health care, and environmental factors from summarized statistical data extracted from the 2018 Robert Wood Johnson Foundation County Health Rankings and merged with US Census data from each of 3138 US counties. The explanatory power of the linear multivariate regression and the top performing machine learning model were compared using mean *R*^2^ measured in 30-fold cross validation.

**Exposures:**

County-level demographic factors (population; rural status; census region; and race/ethnicity, sex, and age composition), socioeconomic factors (median income, unemployment rate, and percentage of population with some college education), health care factors (rate of uninsured adults and primary care physicians), and environmental factors (access to healthy foods and access to exercise opportunities).

**Main Outcomes and Measures:**

County-level obesity prevalence in 2018, its association with each county-level factor, and the percentage of variation in county-level obesity prevalence explained by linear multivariate and gradient boosting machine regression measured with *R*^2^.

**Results:**

Among the 3138 counties studied, the mean (range) obesity prevalence was 31.5% (12.8%-47.8%). In multivariate regressions, demographic factors explained 44.9% of variation in obesity prevalence; socioeconomic factors, 33.0%; environmental factors, 15.5%; and health care factors, 9.1%. The county-level factors with the strongest association with obesity were census region, median household income, and percentage of population with some college education. *R*^2^ values of univariate regressions of obesity prevalence were 0.238 for census region, 0.218 for median household income, and 0.160 for percentage of population with some college education. Multivariate linear regression and gradient boosting machine regression (the best-performing machine learning model) of obesity prevalence using all county-level demographic, socioeconomic, health care, and environmental factors had *R*^2^ values of 0.58 and 0.66, respectively (*P* < .001).

**Conclusions and Relevance:**

Obesity prevalence varies significantly between counties. County-level demographic, socioeconomic, health care, and environmental factors explain the majority of variation in county-level obesity prevalence. Using machine learning models may explain significantly more of the variation in obesity prevalence..

## Key Points

**Question:**

Which factors are associated with county-level variation in obesity prevalence, and how can they be identified using epidemiologic and machine learning methods?

**Findings:**

This cross-sectional study of 3138 US counties found significant county-level variation in obesity prevalence, with US Census region, median household income, and percentage of population with some college education being most strongly associated with obesity prevalence. Machine learning models explain two-thirds more variation in obesity but were less interpretable than multivariate linear regression models.

**Meaning:**

Machine learning models of county-level demographic, socioeconomic, health care, and environmental factors explain significantly more variation in obesity prevalence while being less interpretable.

## Teaser

This cross-sectional study uses summarized statistical data and US Census data to compare epidemiologic and machine learning methods to examine associations of US county-level demographic, socioeconomic, health care, and environmental factors with regional variance in obesity prevalence.

## Introduction

Obesity, defined as body mass index (BMI, calculated as weight in kilograms divided by height in meters squared) greater than 30, is a leading risk factor for and contributor to morbidity and mortality.^1,2^ Prior research has suggested that the obesity epidemic is linked to cardiovascular disease, cancer, and premature mortality. Geographic disparities in obesity prevalence have been documented and associated with demographic, urbanization, socioeconomic, health care, and environmental factors.^3,4,5,6,7^ The Centers for Disease Control and Prevention (CDC) has updated statistics on obesity prevalence by age, education, and state.^8^ The Robert Wood Johnson Foundation County Health Rankings (CHR)^9^ used these and other data to interpolate 2018 county-level information. These data make it possible to create statistical models of how county-level factors are associated with obesity prevalence.

Obesity is a multifactorial problem resulting from individual, community, and geographic influences.^4,10,11^ To better inform public health strategies to combat the obesity epidemic, it is important to understand how county-level factors are associated with obesity prevalence. Previous studies have used traditional epidemiologic methods and factors to explore geographic disparities in obesity.^4,5^ Machine learning has been proposed as an appealing alternative approach for building models of obesity with more predictive power than linear regressions. A trade-off of most machine learning models is that they are based on mathematical functions that do not have readily interpretable variable coefficients.^7,12,13,14^ Our objective was to determine which factors best explain county-level variation in 2018 obesity prevalence and whether traditional epidemiologic methods or machine learning methods are better suited for doing so.

## Methods

### Data Sources

The Strengthening the Reporting of Observational Studies in Epidemiology (STROBE) reporting guidelines for cross-sectional studies were followed by this study. We used data from the 2018 Robert Wood Johnson Foundation CHR.^9^ The CHR is an annually produced county-level data set based on a statistical compilation and interpolation of data from the Behavioral Risk Factor Surveillance System, the Dartmouth Institute, American Community Survey, CDC Diabetes Interactive Atlas, CDC WONDER mortality data, Centers for Medicare & Medicaid Services National Provider Identification, US Census, US Department of Agriculture Food Environment Atlas, and the US Department of Education. Details of data sources considered appear in eTable 1 in the Supplement. The CHR annual county-level rate of obesity is the interpolated county-level percentage of survey respondents whose Behavioral Risk Factor Surveillance System self-reported height and weight correspond to a BMI of 30 or greater.^2,9^ The CHR county-level factors include demographic (population, percentage rural, percentage female, percentage younger than 18 years, percentage 65 years and older, percentage African American, percentage Hispanic, percentage Asian, percentage American Indian/Alaskan Native, and percentage Native Hawaiian/Other); socioeconomic (median household income, percentage of children in poverty, percentage with some college education, percentage food insecure, percentage unemployed, and percentage with severe housing problems); health care (percentage of adults uninsured and primary care provider rate); and environmental factors (percentage with access to exercise opportunities and food environment index) factors. The CHR data were merged with US Census data to identify each county’s census region. A detailed list of the factors, their definitions, and the original data sources on which they are based is included in eTable 1 in the Supplement. This study was based on publicly available and unidentifiable data; thus, Stanford’s institutional review board determined it exempt from review and waived consent.

### Statistical Analysis

Discrepancies in county names were reconciled using the latest US Census data. Counties missing data for a factor considered in our evaluations were omitted from the training and testing of the linear regression models but included in the training and testing of machine learning algorithms, such as gradient boosting machine (GBM) regression, that allow for missing data. For each pair of county-level factors that had a pairwise linear correlation greater than or equal to 0.8, the one with the weaker association with obesity prevalence, as measured by a univariate regression, was excluded. We excluded factors whose association with obesity would have been rendered uninterpretable by endogeneity (ie, if the errors in the estimates of those factors were likely to be correlated with the errors in the estimate of the county-level obesity prevalence).^15^ These were county-level factors whose values were estimated from the same surveys used to estimate obesity prevalence (Behavioral Risk Factor Surveillance System). To reduce the influence of outliers and improve the interpretability of the regression coefficients, continuous variables with values significantly greater than 100 and skewed distributions (eg, population) were log normalized and then scaled to have maximum values of 100. Details of county name changes, counties with missing data, data exclusions, and data normalization are provided in eTable 2 in the Supplement.

Univariate linear regression models were used to determine the association between county-level obesity prevalence and each prespecified individual factor. Multivariate linear regression models were used to find the association between county-level obesity prevalence and all county-level factors in each group of factors: demographic, socioeconomic, health care, and environmental. Multivariate linear regression models were used to find the association between county-level obesity prevalence and all of the factors in all 4 of the above groups. The distributions of obesity prevalence associated with different census regions were compared using the Kolmogorov-Smirnov test with multitest correction.

We compared the percentage of variation in obesity prevalence explained by several machine learning models using all demographic, socioeconomic, health care, and environmental factors. The models were GBM; regression trees; random forest; a linear model chosen using Akaike information criterion, Bayesian information criterion, and their variants from among all models including each factor and each second-order interaction between factors; and a penalized linear model chosen using elastic net variants of the least absolute shrinkage and selection operator (LASSO) from among all models including each available factor and each second order interaction between factors.^16,17,18,19^ To balance underfitting and overfitting (ie, bias and variance), the parameters of each model were tuned using 5-fold cross validation on a training data set of 1000 counties randomly selected from the original data. The training data were divided into 5 subsets or folds. For each parameter of each model, all combinations of values from a predetermined range were combined into a search grid from which values were sampled sequentially. For example, for GBM, the parameters and their ranges were 11 values for the number of trees (150, 160, 170 . . . 250); 6 values for interaction depth (10, 12, 14 . . . 20); 5 values for shrinkage (0.01, 0.02...0.05); and 5 values for N minimum observations in node (2, 4, 6 . . . 10) for a total of 1650 (11 × 6 × 5 × 5) combinations of parameter values (see eTable 3, eFigure 1, and eFigure 2 in the Supplement for details of the parameter tuning of the other models). For each combination of parameter values in the grid, 1 testing fold was selected to be held out, the model was trained on the other 4 folds of the data, and the *R*^2^ was evaluated on the testing fold. This was repeated 5 times for each testing fold, and the average of the *R*^2^ values over the 5 testing folds was reported (ie, no model was tested on the data on which it had been trained). The top performing model and its parameters were selected based on mean *R*^2^.

### Comparison of Linear and Top Performing Models

The amount of variation in obesity prevalence explained by demographic, socioeconomic, health care, and environmental factors using linear regression and the top-performing machine learning model was compared using 30-fold cross validation. For all of the 30 held-out data sets, the resulting *R*^2^ values were compared using the paired Wilcoxon signed-rank test, the nonparametric alternative to the paired *t* test. To test whether additional county-level factors beyond those described above explained more of the variation in obesity prevalence, the above comparison was repeated for all variables available in the data set. All analyses were performed using R version 3.5.1; RStudio Version 1.0.143; and caret, a statistical package for R (The R Foundation).^20^ Statistical significance was determined using 2-sided *P* < .05.

## Results

**Figure 1.**
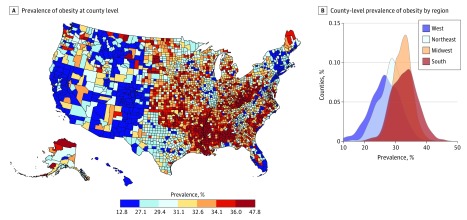
Distribution of Obesity Prevalence by County and Census Region A, Map of US counties by obesity prevalence. B, Density plot of county-level obesity prevalence in each US Census region.

Among the 3138 counties studied, the mean (range) obesity prevalence was 31.5% (12.8%-47.8%) (Figure 1A). The 25th percentile of the 2018 county-level obesity prevalence was 28.8%, the 50th was 31.8%, and the 75th percentile was 34.4%. The South census region had a mean obesity prevalence of 32.9%, the Midwest had a mean prevalence of 32.2%, Northeast had a mean prevalence of 28.6%, and the West had a mean prevalence of 26.6%. The distribution of obesity prevalence differed (*P* < .001) between regions (Figure 1B).

The greatest variation in county-level obesity prevalence, as measured by *R*^2^ in univariate regression, was explained by census region (23.8%), the normalized median household income (21.8%), and percentage of population with some college education (16.0%). Details of univariate regressions for these and all other factors appear in Table 1. In multivariate regressions, demographic factors explained 44.9% of variation in obesity prevalence; socioeconomic factors, 33.0%; environmental factors, 15.5%; and health care factors, 9.1% (Table 2). Multivariate linear regression and gradient boosting machine regression (the best-performing machine learning model) of obesity prevalence using all county-level demographic, socioeconomic, health care, and environmental factors had *R*^2^ values of 58.0% and 66.0%, respectively (*P* < .001). The changes in obesity prevalence associated with a 1 percentage point or 1 unit change in each factor, when controlling for all other factors, are shown in Table 3.

**Table 1.** Variables Included in the Regression Analysis With Summary Statistics and Univariate Regression Results for 2018 County-Level Obesity Prevalence

Variable	Summary Statistics, Mean (SD) [Range], %	Univariate Regression Results	
		Coefficient (SE)	*R*^2^
Demographic factors			
Population	59.1 (10.4) [16.7-100]	−0.0721 (0.0076)^a^	0.0277
Rural	58.6 (31.5) [0-100]	0.0345 (0.0025)^a^	0.0579
Female	49.9 (2.3) [27.8-56.5]	0.1195 (0.0354)^a^	0.0036
Aged <18 y	22.3 (3.5) [0-40.9]	0.2495 (0.0228)^a^	0.0369
Aged ≥65 y	18.4 (4.6) [4.6-56.3]	−0.0624 (0.0176)^a^	0.0040
African American	9.0 (14.3) [0-85.2]	0.1042 (0.0053)^a^	0.1092
Hispanic	9.3 (13.7) [0.5-96.3]	−0.0946 (0.0057)^a^	0.0820
Asian	1.5 (2.9) [0-44.3]	−0.5008 (0.0268 ^a^	0.1005
American Indian/Alaskan Native	2.3 (7.7) [0-93.1]	0.0568 (0.0105)^a^	0.0093
Native Hawaiian/other	0.1 (1.0) [0-50]	−0.3945 (0.0815)^a^	0.0074
Census region			0.2377
Midwest	NA	32.2 (3.0)^a^^,^^b^	NA
Northeast		28.6 (4.0)^a^^,^^b^	
South		32.9 (4.2)^a^^,^^b^	
West		32.4 (4.8)^a^^,^^b^	
Socioeconomic factors			
Household income^c^	91.3 (2.1) [84.7-100]	−1.0254 (0.0347)^a^	0.2179
Some college	57.2 (11.6) [15.5- 94.0]	−0.1563 (0.0064)^a^	0.1597
Food insecure	14.1 (4.2) [3.4- 37.9]	0.4065 (0.0176)^a^	0.1455
Unemployed	5.3 (1.9) [1.7- 23.5]	0.6532 (0.0412)^a^	0.0743
Severe housing problems	14.5 (4.8) [2.7-70.1]	−0.1626 (0.0166)^a^	0.0297
Health care factors			
Uninsured	12.0 (5.1) [2.1-37.4]	−0.0571 (0.0158)^a^	0.0041
Primary care physician rate^c^	12.1 (7.7) [0-100]	−0.1769 (0.0102)^a^	0.0907
Environmental factors			
Access to exercise opportunities	63.0 (23.2) [0-100]	−0.0694 (0.0033)^a^	0.1269
Food environment index	7.4 (1.2) [0-10.0]	−1.0379 (0.0657)^a^	0.0741

Abbreviation: NA, not applicable because variables were not used in the corresponding mode.

^a^ *P* < .01.

^b^ Mean (SD) reported.

^c^ Variables were log normalized and scaled to have a maximum value of 100.

**Table 2.** Multivariate Regression Results

Variable	Demographic Factors^a^	Socioeconomic Factors^b^	Health Care Factors^c^	Environmental Factors^d^	Combined^e^
Observations, No.	3135	3137	3003	3117	2984
*R*^2^	0.452	0.331	0.092	0.156	0.603
Adjusted *R*^2^	0.449	0.330	0.091	0.155	0.600

^a^ Demographic factors include percentage of population, percentage rural, percentage female, percentage younger than 18 years, percentage 65 years and older, percentage African American, percentage Hispanic, percentage Asian, percentage American Indian/Alaskan Native, and percentage Native Hawaiian/other.

^b^ Socioeconomic factors include household income, percentage of children in poverty, percentage with some college, percentage food insecure, percentage unemployed, and percentage with severe housing problems.

^c^ Health care factors include percentage uninsured and primary care physician rate.

^d^ Environmental factors include percentage with access to exercise opportunities and food environment index.

^e^ Combined includes all factors.

**Table 3.** Multivariate Regression

Variable	Coefficient (SE), %				
	Model 1	Model 2	Model 3	Model 4	Model 5^a^
Demographic factors					
Population	0.007 (0.010)				0.004 (0.010)
Rural	0.018 (0.003)^b^				−0.005 (0.003)
Female	−0.170 (0.034)^b^				0.031 (0.034)
Aged <18 y	0.351 (0.029)^b^				0.297 (0.028)^b^
Aged ≥65 y	0.016 (0.023)				−0.080 (0.021)^b^
African American	0.082 (0.005)^b^				0.055 (0.006)^b^
Hispanic	−0.073 (0.005)^b^				−0.071 (0.006)^b^
Asian	−0.256 (0.025)^b^				−0.047 (0.027)
American Indian/Alaskan Native	0.057 (0.009)^b^				0.076 (0.010)^b^
Native Hawaiian/other	0.160 (0.064)^c^				0.307 (0.145)^c^
Census region					
Northeast	−1.876 (0.271)^b^				−1.777 (0.242)^b^
South	0.184 (0.163)				−0.473 (0.166)^b^
West	−4.390 (0.208)^b^				−3.899 (0.199)^b^
Socioeconomic factors					
Household income		−0.340 (0.052)^b^			−0.667 (0.051)^b^
Some college		−0.073 (0.008)^b^			−0.077 (0.008)^b^
Food insecure		0.310 (0.023)^b^			−0.017 (0.033)
Unemployed		0.151 (0.045)^b^			0.199 (0.039)^b^
Severe housing problems		−0.305 (0.016)^b^			−0.156 (0.017)^b^
Health care factors					
Uninsured			0.003 (0.016)		−0.139 (0.017)^b^
Primary care physician rate			−0.177 (0.010)^b^		−0.044 (0.008)^b^
Environmental factors					
Access to exercise opportunities				−0.687 (0.066)^b^	−0.009 (0.003)^b^
Food environment index				−0.058 (0.003)^b^	0.025 (0.088)
Observations, No.	3135	3137	3003	3117	2984
*R*^2^	0.452	0.331	0.092	0.156	0.603
Adjusted *R*^2^	0.449	0.330	0.091	0.155	0.600

Abbreviation: SE, standard error.

^a^ Combined category includes all factors.

^b^ *P* < .01.

^c^ *P* < .05.

### Comparison of Machine Learning Regression Models

Gradient boosting machine outperformed random forest, regression tree, and models selected using variants of the Akaike information criterion, Bayesian information criterion, and LASSO as measured by *R*^2^ in 5-fold cross validation. The top performing model was GBM, with an *R*^2^ of 0.65. The model with the next best performance was LASSO, with all second-order variable interactions, with an *R*^2^ of 0.64. The parameters of the GBM model with the highest *R*^2^ were number of trees = 180, interaction depth = 20; shrinkage = 0.05, and minimum number of observations in node = 8. See eFigure 1 and eFigure 2 in the Supplement for the performance of GBM and LASSO for a variety of parameter settings, eTable 3 in the Supplement for the top performance and the corresponding parameters of each of the models considered, and eTable 3 in the Supplement for the relative importance of the variables in the GBM model.

### Comparison of Linear Multivariate and GBM Regression Models

**Figure 2.**
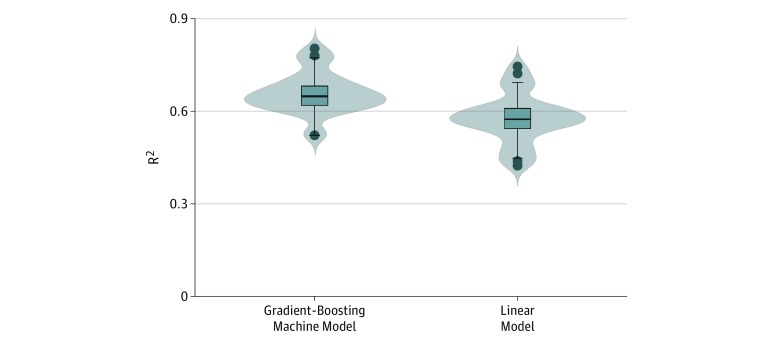
Comparison of Performance of Gradient Boosting Machine Regression and Linear Multivariate Regression Using 30-Fold Cross Validation Violin plots of the distribution of the *R*^2^ values of the gradient boosting machine and linear model models. The box plots inside the violin plot show the following values of the distribution of *R*^2^ for the gradient boosting machine and linear models: the middle lines indicate the medians, the bottom and top of each box show the 25th and 75th percentiles, respectively, the bottom whiskers show the values of the 25th percentile minus 1.5 × the interquartile range, the top whiskers show the values of the 75th percentile plus 1.5 × the interquartile range, and the top and bottom points are all outliers, defined as points in the data that lie below and above the whiskers.

When trained on all demographic, socioeconomic, environmental, and health care access factors and tested on new data, the linear multivariate and GBM regression explained 58.1% and 66.1% (*P* < .001) of the variation of obesity prevalence, respectively (Figure 2). The addition of county-level factors beyond those described led to small mean increases in the percentage of variation explained by each model, significant for the linear model and not significant for the GBM model (eTable 4 in the Supplement).

## Discussion

Using 2018 national county-level data, we found that county-level obesity prevalence showed significant geographic heterogeneity, and that this was largely explained by county-level demographic, socioeconomic, health care, and environmental factors. Using traditional epidemiologic approaches, these factors explained 58% of the variation in obesity prevalence at the county level. Using a machine learning approach, these factors explained two-thirds of the variation.

Demographic and socioeconomic factors explained a significant percentage of the variation in county-level obesity. The individual factors that explained the greatest percentage of variation were census region (North, South, West, Midwest), median household income, and percentage of population with some college education. These findings are consistent with previous studies that have identified significant geographic disparities in obesity prevalence.^4,10^ In particular, the South has been strongly positively associated with obesity prevalence.^10^ Census region still had significant explanatory statistical power after adjusting for all available factors. This suggests substantive differences in regional obesity prevalence well beyond those explained by demographic or socioeconomic factors. The association between county-level obesity prevalence and median household income and percentage of population with some college education accords with studies documenting an inverse relationship between socioeconomic status and obesity prevalence.^3,4,6^ There are socioeconomic differences in engaging in physical activity that are associated in part to access to recreational resources and perceived safety of neighborhood.^21,22^

Our finding that the percentage of African American individuals in the population explained more than 10% of the variation in county-level obesity is noteworthy and concordant with other studies.^4,10,23^ This is associated with the higher proportion of African American individuals living in the South and counties with lower median income, although it remains an important independent predictor of obesity.^4^ Counties with higher proportions of African American individuals may have fewer healthy food options and poorer opportunities for physical activity.^24,25^ On the other hand, there was a negative association between the percentage of Hispanic persons in a county and obesity prevalence, despite Hispanic persons having greater obesity rates compared with other racial/ethnic groups. Previous county-level studies^13^ have also documented this negative association, but an increase in Hispanic population has been associated with an increase in obesity prevalence.^23^ Some have speculated that this may be because Hispanic populations are dense in regions associated with lower obesity prevalence.^4,10^

Our study is complementary to and extends earlier literature by showing that machine learning may be used to explain more variation in county-level obesity prevalence than traditional epidemiologic models.^7,12,13,14^ To our knowledge, this is the first study to analyze county-level national data using machine learning algorithms. Our top-performing machine learning model explained two-thirds of the variation in county-level obesity prevalence, significantly more than traditional multivariate linear models.

Epidemiologic approaches including limited, preselected variables may offer interpretable results. We found that including machine learning approaches significantly improved the total amount of variation in obesity prevalence and improved estimates of obesity prevalence in counties about which this information is unavailable. When weighing the interpretability of linear regression for decision making against the performance of machine learning models, 3 factors should be considered. First, multivariate regression models may appear more interpretable than they are, for example, owing to confounding variables. Second, machine learning algorithms offer partially interpretable outputs, such as variable importance (eFigure 3 in the Supplement). Third, some machine learning models offer both superior performance and interpretability on par with that of multivariate linear regression. Our second-best performing model, LASSO with all second-order interactions, is substantially simpler than GBM and achieved similar performance. The take-home message from these considerations is that for some decisions there may be more benefits and fewer drawbacks to using powerful machine learning models.

Each of our models, including the linear regression, had significantly higher performance on the data on which they were trained than on the data on which they were tested. This demonstrates the importance of evaluating performance on testing data not previously seen by the model. We measured the percentage of variation explained using 30-fold cross validation. In particular, there were 30 repetitions of training each model on training data and testing it on entirely separate testing data. This ensures that performance is greater for models that identify relationships that exist in the data rather than models that overfit the data with spurious mathematical relationships. Our approach contrasts with the common practice of fitting a single model to the data and reporting the performance of the model (eg, *R*^2^) only on the data on which it was fit.

### Limitations

Our findings should be interpreted in light of several limitations. Our analysis is based on CHR data, many of the fields of which are self-reported, sampled randomly from the population, and interpolated using statistical methods. It is likely that self-reported obesity underestimates obesity prevalence.^26,27^ If this bias is nondifferential by county or other factors considered, our statistical results remain directionally valid. Furthermore, obesity prevalence was based on BMI, which is an indirect measure of adiposity and health risk. At the same BMI level, non-Hispanic African American adults have lower adiposity compared with non-Hispanic white adults.^28^ Health risks begin at a lower BMI among Asian adults than among non-Hispanic white adults.^29^ Therefore, BMI is an indirect measure of the health risks associated with increased adiposity. Our analyses and conclusions are restricted to the variables that are routinely captured in these data sets. Individual-level risk is not accounted for. Owing to the nature of the mathematical models underlying machine learning algorithms, such models do not produce readily interpretable variable coefficients. They do not establish causal relationships or make clear the reasons certain predictors are more important than others.

## Conclusions

County-level demographic, socioeconomic, health care, and environmental factors explain the majority of the variation in county-level obesity prevalence. Machine learning models explain significantly more of the variation in obesity prevalence than traditional models. For decisions about obesity prevalence based on population characteristics, there may be more benefits and fewer drawbacks to using powerful machine learning models.

## Notes

Supplement. **eTable 1.** Definition of Variables **eTable 2.** Data Changes and Exclusions **eTable 3.** The Machine Learning Models Considered, Their Parameters of, Parameter Values for the Top Performing Setting, and Performance as Measured by *R*^2^ Averaged Over 5-Fold Cross Validation **eTable 4.** Results of Performance Comparison Between Linear Regression and Gradient Boosting Machine Regression for Selected Variables and All Available Variables Using 30-Fold Cross Validation **eFigure 1.** The Performance of the GBM Model, as Measured by *R*^2^ Averaged Over 5-Fold Cross Validation, Plotted for a Variety of Parameter Settings **eFigure 2.** The Performance of the LASSO Model, as Measured by *R*^2^ Averaged Over 5-Fold Cross Validation, Plotted for a Variety of Parameter Settings. **eFigure 3.** Relative Variable Importance for GBM Over 30 Folds of Cross Validation

## References

[1] Visscher TLS, Seidell JC The public health impact of obesity. Annu Rev Public Health. 2001;22(1):-. doi:10.1146/annurev.publhealth.22.1.355 11274526

[2] Stokes A, Preston SH Deaths attributable to diabetes in the United States: comparison of data sources and estimation approaches. PLoS One. 2017;12(1):e0170219. doi:10.1371/journal.pone.0170219 28121997 PMC5266275

[3] Dwyer-Lindgren L, Freedman G, Engell RE, Prevalence of physical activity and obesity in US counties, 2001-2011: a road map for action. Popul Health Metr. 2013;11(1):7. doi:10.1186/1478-7954-11-7 23842197 PMC3718620

[4] Myers CA, Slack T, Martin CK, Broyles ST, Heymsfield SB Regional disparities in obesity prevalence in the United States: a spatial regime analysis. Obesity (Silver Spring). 2015;23(2):481-487. doi:10.1002/oby.20963 25521074 PMC4310761

[5] von Hippel P, Benson R Obesity and the natural environment across US counties. Am J Public Health. 2014;104(7):1287-1293. doi:10.2105/AJPH.2013.301838 24832148 PMC4056217

[6] Hales CM, Fryar CD, Carroll MD, Freedman DS, Aoki Y, Ogden CL Differences in obesity prevalence by demographic characteristics and urbanization level among adults in the United States, 2013-2016. JAMA. 2018;319(23):2419-2429. doi:10.1001/jama.2018.7270 29922829 PMC6583043

[7] Maharana A, Nsoesie EO Use of deep learning to examine the association of the built environment with prevalence of neighborhood adult obesity. JAMA Netw Open. 2018;1(4):e181535. doi:10.1001/jamanetworkopen.2018.1535 30646134 PMC6324519

[8] Centers for Disease Control and Prevention New adult obesity maps. https://www.cdc.gov/obesity/data/prevalence-maps.html. Published August 30, 2017. Accessed July 9, 2018.

[9] Remington PL, Catlin BB, Gennuso KP The County Health Rankings: rationale and methods. Popul Health Metr. 2015;13(1):11. doi:10.1186/s12963-015-0044-2 25931988 PMC4415342

[10] Slack T, Myers CA, Martin CK, Heymsfield SB The geographic concentration of US adult obesity prevalence and associated social, economic, and environmental factors. Obesity (Silver Spring). 2014;22(3):868-874. doi:10.1002/oby.20502 23630100 PMC13722265

[11] Gurka MJ, Filipp SL, DeBoer MD Geographical variation in the prevalence of obesity, metabolic syndrome, and diabetes among US adults. Nutr Diabetes. 2018;8(1):14. doi:10.1038/s41387-018-0024-2 29549249 PMC5856741

[12] Golino HF, Amaral LS de B, Duarte SFP, Predicting increased blood pressure using machine learning. J Obes. 2014;2014:637635. doi:10.1155/2014/637635 24669313 PMC3941962

[13] DeGregory KW, Kuiper P, DeSilvio T, A review of machine learning in obesity. Obes Rev. 2018;19(5):668-685. doi:10.1111/obr.12667 29426065 PMC8176949

[14] Dugan TM, Mukhopadhyay S, Carroll A, Downs S Machine learning techniques for prediction of early childhood obesity. Appl Clin Inform. 2015;6(3):506-520. doi:10.4338/ACI-2015-03-RA-0036 26448795 PMC4586339

[15] Angrist JD, Pischke J-S Mostly Harmless Econometrics: An Empiricist’s Companion. Princeton, NJ: Princeton University Press; 2008. doi:10.2307/j.ctvcm4j72

[16] Friedman JH Greedy function approximation: a gradient boosting machine. Ann Stat. 2001;29(5):1189-1232. doi:10.1214/aos/1013203451

[17] Breiman L Random forest. Mach Learn. 2017;45(1):5-32. doi:10.1023/A:1010933404324

[18] Neath AA, Cavanaugh JE The Bayesian information criterion: background, derivation, and applications. Wiley Interdiscip Rev Comput Stat. 2011;4(2):199-203. doi:10.1002/wics.199

[19] Zou H, Hastie T Regularization and variable selection via the elastic net. J R Stat Soc Series B Stat Methodol. 2005;67(2):301-320. doi:10.1111/j.1467-9868.2005.00503.x

[20] Kuhn M Building predictive models in R using the caret package. J Stat Softw. 2008;28(5). doi:10.18637/jss.v028.i05

[21] Edwards MB, Jilcott SB, Floyd MF, Moore JB County-level disparities in access to recreational resources and associations with adult obesity. J Park Recreat Admi. 2011;29(2):39-54.

[22] Kamphuis CB, van Lenthe FJ, Giskes K, Huisman M, Brug J, Mackenbach JP Socioeconomic differences in lack of recreational walking among older adults: the role of neighbourhood and individual factors. Int J Behav Nutr Phys Act. 2009;6(1):1. doi:10.1186/1479-5868-6-1 19123927 PMC2631001

[23] Myers CA, Slack T, Martin CK, Broyles ST, Heymsfield SB Change in obesity prevalence across the United States is influenced by recreational and healthcare contexts, food environments, and hispanic populations. PLoS One. 2016;11(2):e0148394. doi:10.1371/journal.pone.0148394 26849803 PMC4743954

[24] Singleton CR, Affuso O, Sen B Decomposing racial disparities in obesity prevalence: variations in retail food environment. Am J Prev Med. 2016;50(3):365-372. doi:10.1016/j.amepre.2015.08.004 26507301 PMC4762716

[25] Congdon P Variations in obesity rates between US counties: impacts of activity access, food environments, and settlement patterns. Int J Environ Res Public Health. 2017;14(9):E1023. doi:10.3390/ijerph14091023 28880209 PMC5615560

[26] Ward ZJ, Long MW, Resch SC, Redrawing the US obesity landscape: bias-corrected estimates of state-specific adult obesity prevalence. PLoS One. 2016;11(3):e0150735. doi:10.1371/journal.pone.0150735 26954566 PMC4782996

[27] Connor Gorber S, Tremblay M, Moher D, Gorber B A comparison of direct vs. self-report measures for assessing height, weight and body mass index: a systematic review. Obes Rev. 2007;8(4):307-326. doi:10.1111/j.1467-789X.2007.00347.x 17578381

[28] Heo M, Faith MS, Pietrobelli A, Heymsfield SB Percentage of body fat cutoffs by sex, age, and race-ethnicity in the US adult population from NHANES 1999-2004. Am J Clin Nutr. 2012;95(3):594-602. doi:10.3945/ajcn.111.025171 22301924

[29] Zheng W, McLerran DF, Rolland B, Association between body-mass index and risk of death in more than 1 million Asians. N Engl J Med. 2011;364(8):719-729. doi:10.1056/NEJMoa1010679 21345101 PMC4008249

