# Supplementary material for: Identification of Factors Associated With Variation in US County-Level Obesity Prevalence Rates Using Epidemiologic vs Machine Learning Models
Source: JAMA Netw Open. 2019 Apr 26;2(4):e192884. doi: 10.1001/jamanetworkopen.2019.2884 (PMC6487629; doi:10.1001/jamanetworkopen.2019.2884)
Supplement: Supplement. — eTable 1. Definition of Variables eTable 2. Data Changes and Exclusions eTable 3. The Machine Learning Models Considered, Their Parameters of, Parameter Values for the Top Performing Setting, and Performance as Measured by R2 Averaged Over 5-Fold Cross Validation eTable 4. Results of Performance Comparison Between Linear Regression and Gradient Boosting Machine Regression for Selected Variables and All Available Variables Using 30-Fold Cross Validation eFigure 1. The Performance of the GBM Model, as Measured by R2 Averaged Over 5-Fold Cross Validation, Plotted for a Variety of Parameter Settings eFigure 2. The Performance of the LASSO Model, as Measured by R2 Averaged Over 5-Fold Cross Validation, Plotted for a Variety of Parameter Settings. eFigure 3. Relative Variable Importance for GBM Over 30 Folds of Cross Validation [file jamanetwopen-2-e192884-s001.pdf]

Supplementary Online Content

Scheinker D, Valencia A, Rodriguez F. Identification of factors associated with variation in US county-level obesity prevalence rates using epidemiologic vs machine learning models. *JAMA Netw Open*. 2019;2(4):e192884. doi:10.1001/jamanetworkopen.2019.2884

eTable 1. Definition of Variables

eTable 2. Data Changes and Exclusions

eTable 3. The Machine Learning Models Considered, Their Parameters of, Parameter Values for the Top Performing Setting, and Performance as Measured by R^2 Averaged Over 5-Fold Cross Validation

eTable 4. Results of Performance Comparison Between Linear Regression and Gradient Boosting Machine Regression for Selected Variables and All Available Variables Using 30-Fold Cross Validation

eFigure 1. The Performance of the GBM Model, as Measured by R^2 Averaged Over 5-Fold Cross Validation, Plotted for a Variety of Parameter Settings

eFigure 2. The Performance of the LASSO Model, as Measured by R^2 Averaged Over 5-Fold Cross Validation, Plotted for a Variety of Parameter Settings.

e Figure 3. Relative Variable Importance for GBM Over 30 Folds of Cross Validation

This supplementary material has been provided by the authors to give readers additional information about their work.

© 2019 Scheinker D et al. *JAMA Network Open*.

eTable 1. Definition of Variables

Variables	Description	Sources of Data	Year	Category
**Outcome**				
Adult obesity	Percentage of adults that report a BMI of 30 or more	CDC Diabetes Interactive Atlas	2014	
**Selected Variables**				
Population	Number of persons	Census Population Estimates	2016	Demographics
% Rural	Urban areas are defined as having 50,000 or more people. Rural encompasses all population, housing, and territory not included within an urban area.	Census Population Estimates	2010	Demographics
% Females	Number of females in county	Census Population Estimates	2016	Demographics
% below 18 years of age	Number of persons less than 18 years old	Census Population Estimates	2016	Demographics
% 65 and older	Number of persons at or greater than 65 years old	Census Population Estimates	2016	Demographics
% Non-Hispanic African American	Persons self-identifying as non-Hispanic African-American	Census Population Estimates	2016	Demographics
% Hispanic	Persons self-identifying as Hispanic	Census Population Estimates	2016	Demographics
% Asian	Persons self-identifying as Asian	Census Population Estimates	2016	Demographics
% American Indian and Alaskan Native	Persons self-identifying as American Indian/Alaskan Native	Census Population Estimates	2016	Demographics
% Native Hawaiian/Other Pacific Islander	Persons self-identifying as Native Hawaiian/Other	Census Population Estimates	2016	Demographics
Region	Census regions are groupings of states and the District of Columbia that subdivide the United States for the presentation of census data. The Census Bureau defines four census regions and identifies each one with a single-digit census code- Northeast (1), Midwest (2), South (3), and West (4)	US Census	2016	Demographics

© 2019 Scheinker D et al. *JAMA Network Open*.

Median household income	Median Household Income is the income where half of households in a county earn more and half of households earn less.	Small Area Income and Poverty Estimates	2016	Socioeconomic
Some college	Percentage of adults ages 25-44 with some post-secondary education	American Community Survey, 5-year estimates	2012-2016	Socioeconomic
Food Insecurity	Food Insecurity is the percentage of the population who did not have access to a reliable source of food during the past year.	Map the Meal Gap	2015	Socioeconomic
Unemployment	Percentage of population ages 16 and older unemployed but seeking work	Bureau of Labor Statistics	2016	Socioeconomic
Severe housing problems	Percentage of households with at least 1 of 4 housing problems: overcrowding, high housing costs, or lack of kitchen or plumbing facilities	Comprehensive Housing Affordability Strategy (CHAS) data	2010-2014	Socioeconomic
Uninsured	Percentage of population under age 65 without health insurance	Small Area Health Insurance Estimates	2015	Healthcare
Primary care physicians	Ratio of population to primary care physicians	Area Health Resource File/American Medical Association	2015	Healthcare
Access to exercise opportunities	Percentage of population with adequate access to locations for physical activity	Business Analyst, Delorme map data, ESRI, & US Census Tigerline Files	2010 & 2016	Environmental
Food environment index	Index of factors that contribute to a healthy food environment, 0 (worst) to 10 (best)	USDA Food Environment Atlas, Map the Meal Gap from Feeding America	2015	Environmental
**Additional Variables**				
Children in single-parent households	Percentage of children that live in a household headed by single parent	American Community Survey, 5-year estimates	2012-2016	Additional
Social associations	Number of membership associations per 10,000 population	County Business Patterns	2015	Additional

Drinking water violations	Indicator of the presence of health-related drinking water violations. Yes indicates the presence of a violation, No indicates no violation.	Safe Drinking Water Information System	2016	Additional
Driving alone to work	Percentage of the workforce that drives alone to work	American Community Survey, 5-year estimates	2012-2016	Additional
Long commute - driving alone	Among workers who commute in their car alone, the percentage that commute more than 30 minutes	American Community Survey, 5-year estimates	2012-2016	Additional
Limited access to healthy foods	Limited Access to Healthy Foods is the percentage of the population that is low income and does not live close to a grocery store.	USDA Food Environment Atlas	2015	Additional
Drug overdose deaths	Drug Overdose Deaths are the number of deaths due to drug poisoning per 100,000 population. ICD-10 codes used include X40-X44, X60-X64, X85, and Y10-Y14.	CDC WONDER mortality data	2014-2016	Additional
Insufficient sleep	Insufficient Sleep is the percentage of adults who responded to the following question by stating they sleep less than 7 hours per night: "On average, how many hours of sleep do you get in a 24-hour period?	Behavioral Risk Factor Surveillance System	2016	Additional
Health care costs	Health Care Costs are the price-adjusted Medicare reimbursements (Parts A and B) per enrollee	Dartmouth Atlas of Health Care	2015	Additional
80th Percentile Income	Households with income at 80th percentile	Small Area Income and Poverty Estimates	2016	Socioeconomic
20th Percentile Income	Households with income at 20th percentile	Small Area Income and Poverty Estimates	2016	Socioeconomic
**Excluded Variables**				
Income inequality	Ratio of household income at the 80th percentile to income at the 20th percentile	American Community Survey, 5-year estimates	2012-2016	Excluded*
Residential segregation - black/white	Racial/ethnic residential segregation refers to the degree to which two or more groups live separately from one another in a geographic area.	American Community Survey, 5-year estimates	2012-2016	Excluded*

Residential segregation - non-white/white	Racial/ethnic residential segregation refers to the degree to which two or more groups live separately from one another in a geographic area.	American Community Survey, 5-year estimates	2012-2016	Excluded*
% not proficient in English	Number of respondents using another language at home other than English	American Community Survey, 5-year estimates	2012-2016	Excluded*
Dentists	Ratio of population to dentists	Area Health Resource File/National Provider Identification file	2016	Excluded*
Poor or fair health	Percentage of adults reporting fair or poor health (age-adjusted)	Behavioral Risk Factor Surveillance System	2016	Excluded**
Poor physical health days	Average number of physically unhealthy days reported in past 30 days (age-adjusted)	Behavioral Risk Factor Surveillance System	2016	Excluded**
Poor mental health days	Average number of mentally unhealthy days reported in past 30 days (age-adjusted)	Behavioral Risk Factor Surveillance System	2016	Excluded**
Diabetes	Diabetes prevalence is the prevalence of diagnosed diabetes in a given county.	Behavioral Risk Factor Surveillance System	2014	Excluded**
Adult smoking	Percentage of adults who are current smokers	Behavioral Risk Factor Surveillance System	2016	Excluded**
Excessive drinking	Percentage of adults reporting binge or heavy drinking	Behavioral Risk Factor Surveillance System	2016	Excluded**
Frequent physical distress	Frequent Physical Distress is the percentage of adults who reported ≥14 days in response to the question, "Thinking about your physical health, which includes physical illness and injury, for how many days during the past 30 days was your physical health not good?"	Behavioral Risk Factor Surveillance System	2016	Excluded**
Frequent mental distress	Frequent Mental Distress is the percentage of adults who reported ≥14 days in response to the question, "Now, thinking about your mental health, which includes stress, depression,	Behavioral Risk Factor Surveillance System	2016	Excluded**

	and problems with emotions, for how many days during the past 30 days was your mental health not good?"			
Physical inactivity	Percentage of adults age 20 and over reporting no leisure-time physical activity	CDC Diabetes Interactive Atlas	2014	Excluded**
Injury deaths	Number of deaths due to injury per 100,000 population	CDC WONDER mortality data	2012-2016	Excluded*
Child mortality	Child Mortality is the number of deaths among children under age 18 per 100,000 population.	CDC WONDER mortality data	2013-2016	Excluded*
Infant mortality	Infant Mortality measures the number of deaths among children less than one year of age per 1,000 live births.	CDC WONDER mortality data	2010-2016	Excluded*
Motor vehicle crash deaths	Motor Vehicle Crash Deaths are the number of deaths due to traffic accidents involving a motor vehicle per 100,000 population.	CDC WONDER mortality data	2010-2016	Excluded*
Homicides	Homicides are the number of deaths from assaults, defined as ICD-10 codes X85-Y09, per 100,000 population.	CDC WONDER mortality data	2010-2016	Excluded*
Firearm fatalities	Firearm Fatalities are the number of deaths due to firearms, defined as ICD-10 codes W32-W34, X72-X74, X93-X95, Y22-Y24, and Y35.0, per 100,000 population.	CDC WONDER mortality data	2012-2016	Excluded*
% Non-Hispanic white	Persons self-identifying as non-Hispanic white	Census Population Estimates	2016	Demographics
Mental health providers	Ratio of population to mental health providers	CMS, National Provider Identification	2017	Excluded*
Other primary care providers	Other Primary Care Providers is the ratio of the county population to the number of other primary care providers	CMS, National Provider Identification	2017	Excluded*
Preventable hospital stays	Number of hospital stays for ambulatory-care sensitive conditions per 1,000 Medicare enrollees	Dartmouth Atlas of Health Care	2015	Excluded*
Diabetes monitoring	Percentage of diabetic Medicare enrollees ages 65-75 that receive HbA1c monitoring	Dartmouth Atlas of Health Care	2014	Excluded*
Mammography screening	Percentage of female Medicare enrollees ages 67-69 that receive mammography screening	Dartmouth Atlas of Health Care	2014	Excluded*
High school graduation	Percentage of ninth-grade cohort that graduates in four years	EDFacts	2014-2015	Excluded*

Air pollution - particulate matter[1]	Average daily density of fine particulate matter in micrograms per cubic meter (PM2.5)	Environmental Public Health Tracking Network	2012	Excluded*
Alcohol-impaired driving deaths	Percentage of driving deaths with alcohol involvement	Fatality Analysis Reporting System	2012-2016	Excluded*
Disconnected youth	Disconnected Youth is the percentage of teens and young adults ages 16-24 who are neither working nor in school.	Measure of America	2010-2014	Excluded*
Children eligible for free or reduced price lunch	Children Eligible for Free or Reduced Price Lunch is the percentage of children enrolled in public schools, grades PK - 12, eligible for free (family income less than 130% of federal poverty level) or reduced price (family income less than 185% of federal poverty level) lunch.	National Center for Education Statistics	2015-2016	Excluded*
Drug overdose deaths - modeled	Drug Overdose Deaths are a modeled estimate of the number of deaths due to drug poisoning per 100,000 population. ICD-10 codes used include X40-X44, X60-X64, X85, and Y10-Y14.	National Center for Health Statistics - Data.CDC.gov	2016	Excluded*
Premature death	Years of potential life lost before age 75 per 100,000 population (age-adjusted)	National Center for Health Statistics - Mortality Files	2014-2016	Excluded*
Low birthweight	Percentage of live births with low birthweight (< 2500 grams)	National Center for Health Statistics - Natality files	2010-2016	Excluded*
Teen births	Number of births per 1,000 female population ages 15-19	National Center for Health Statistics - Natality files	2010-2016	Excluded*
HIV Prevalence	HIV prevalence measures the number of diagnosed cases of HIV for persons aged 13 years and older in a county per 100,000 population	National Center for HIV/AIDS, Viral Hepatitis, STD, and TB Prevention	2015	Excluded*
Sexually transmitted infections	Number of newly diagnosed chlamydia cases per 100,000 population	National Center for HIV/AIDS, Viral Hepatitis, STD, and TB Prevention	2015	Excluded*
Uninsured adults	Uninsured Adults is the percentage of the population ages 18 to 64 that has no health insurance coverage in a given county.	Small Area Health Insurance Estimates	2015	Excluded*

Uninsured children	Uninsured Children is the percentage of the population under age 19 that has no health insurance coverage.	Small Area Health Insurance Estimates	2015	Excluded*
Children in poverty	Percentage of children under age 18 in poverty	Small Area Income and Poverty Estimates	2016	Additional
Violent crime	Number of reported violent crime offenses per 100,000 population	Uniform Crime Reporting - FBI	2012-2014	Excluded*

*Excluded due to more than 200 missing values **Excluded to avoid inclusion of coefficients uninterpretable due to endogeneity

eTable 2. Data Changes and Exclusions

Variables	Explanation for exclusion/change
County Name	Kusilvak and Wade Hampton counties in Alaska are the same, but both were listed in the CHR dataset, so Kusilvak was omitted.<br><br>Skagway and Hoonah-Angoon counties in Alaska were combined, and Petersburg, Wrangell, and Prince of Wales-Hyder, and Ketchikan Gateway counties in Alaska were combined.

Include details of county name changes, counties with missing data, data exclusions, and data normalization.

© 2019 Scheinker D et al. *JAMA Network Open*.

eTable 3. The Machine Learning Models Considered, Their Parameters of, Parameter Values for the Top Performing Setting, and Performance as Measured by R^2 Averaged Over 5-Fold Cross Validation

**Model**	**Function & Package**	**Tuning parameters**	**Parameter values considered**	**Parameter value for top performing model**	**R^2 of top performing model**
Regression Trees	Rpart, rpart	Complexity parameter	0.002, 0.004, 0.006, 0.008, 0.01	0.0004	0.43
**Gradient boosting machine**	Gbm, gbm	Interaction depth	10, 12, 14 ...20	20	**0.65**
		Number of trees	150, 160, 170 ...250	180	
		Shrinkage	0.01, 0.02, ...0.05	0.05	
		Minimum observations in node	2, 4, 6 ...10	8	
Random forests	Rf, randomForest	Number of variables available for splitting at each tree node	1, 2, 3, …15	6	0.61
LASSO[+]	Lasso, elasticnet	Lambda – regularization parameter	0.01, 0.02, …0.1	0.04	0.61
		Alpha	0.5, 1, 1.5, 2	0.5	
LASSO with all second order variable interactions[*]	Lasso, elasticnet	Lambda – regularization parameter	0.01, 0.02, …0.1	0.03	0.64
		Alpha	0.5, 1, 1.5, 2	1	
Akaike/Bayesian Information Criteria with all second order variable interactions[#]	step	k – complexity parameter	2, 4, ln(800), 8[!]	ln(800)	0.57

+Regression with variables selected with LASSO. *Regression with variables selected with LASSO from all possible second order interactions of the variables. #Regression with variables selected with backwards stepwise selection starting from a full model including all second order interactions, the settings corresponding to AIC and BIC are, respectively, k=2 and k=log(n) where log is the natural logarithm and n is the number of entries in the training set (e.g., log(800) = 6.68)

eTable 4. Results of Performance Comparison Between Linear Regression and Gradient Boosting Machine Regression for Selected Variables and all Available Variables Using 30-Fold Cross Validation

	**Selected Variables**	**All Variables**	**Model Difference**
Models	Average testing R^2	Mean testing R^2	
Linear Regression	0.58	0.60	0.02 (p = 0.003)
Gradient Boosting Machine (GBM) Regression	0.66	0.68	0.02 (p = 0.140)
Model Differences	0.08 (p-value < 0.001)	0.08 (p-value < 0.001)	

© 2019 Scheinker D et al. *JAMA Network Open.*

eFigure 1. The Performance of the GBM Model, as Measured by R^2 Averaged Over 5-Fold Cross Validation, Plotted for a Variety of Parameter Settings

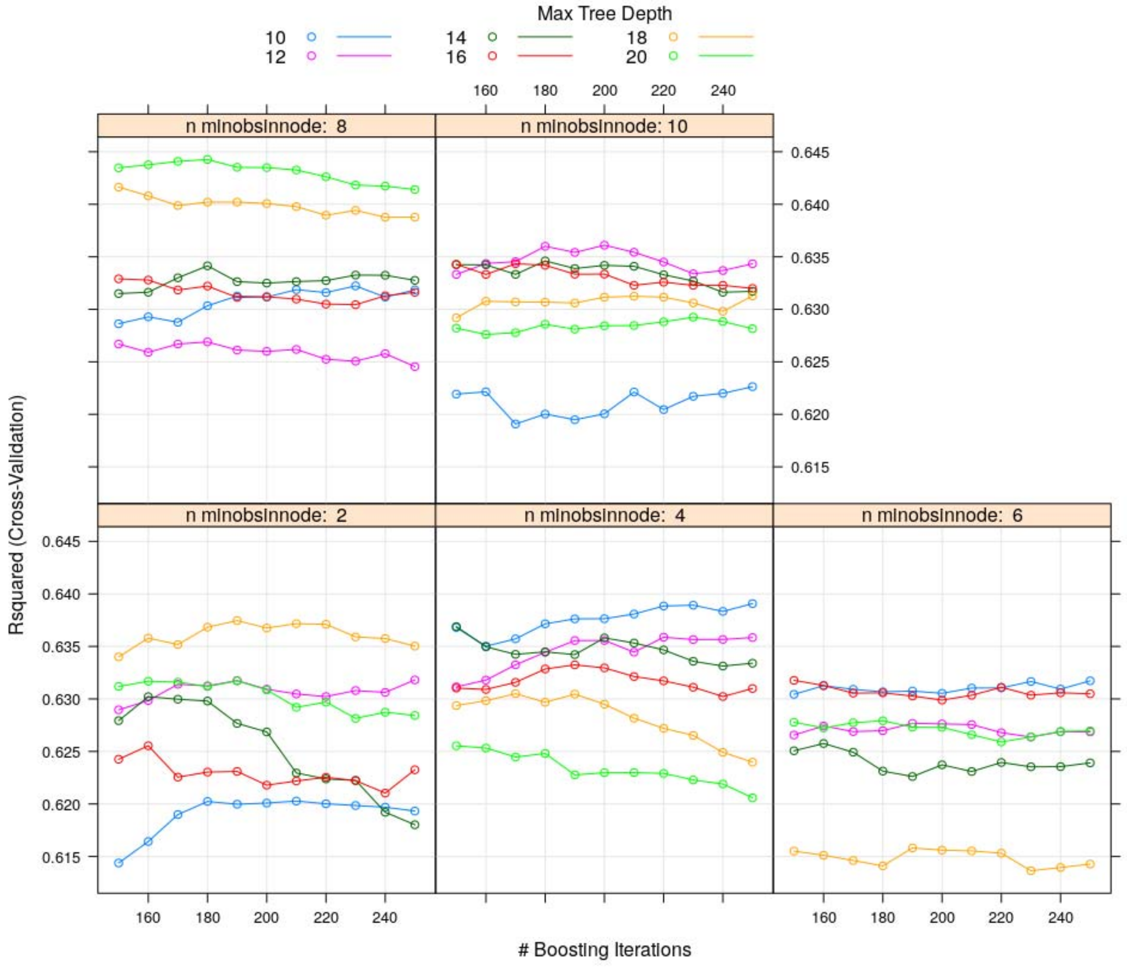

Each plot corresponds to a distinct setting of the parameter Minimum Observations in Node. Each plot shows the mean R^2 calculated with 5-fold cross validation (y-axis) as a function of the settings of the parameter Number of Boosting Iterations. Each line in each plot corresponds to a distinct setting of the parameter Maximum Tree Depth.

© 2019 Scheinker D et al. *JAMA Network Open*.

eFigure 2. The Performance of the LASSO Model, as Measured by R^2 Averaged Over 5-Fold Cross Validation, Plotted for a Variety of Parameter Settings. Overlapping Lines Not Visible

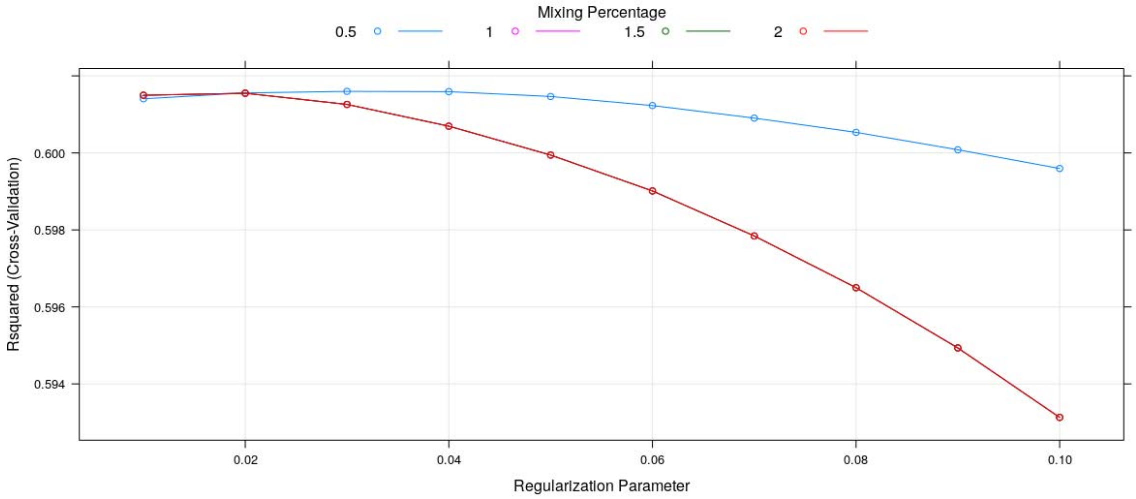

The mean R^2 calculated with 5-fold cross validation (y-axis) is shown as a function of the distinct values of the Regularization Parameter λ. Each line in each plot corresponds to a distinct setting of the mixing parameter α ($\alpha = 1$ corresponds to LASSO). Values of α that resulted in equivalent models result in overlapping lines not visible in the plot.

© 2019 Scheinker D et al. *JAMA Network Open*.

e **Figure 3.** Relative Variable Importance for GBM Over 30 Folds of Cross Validation

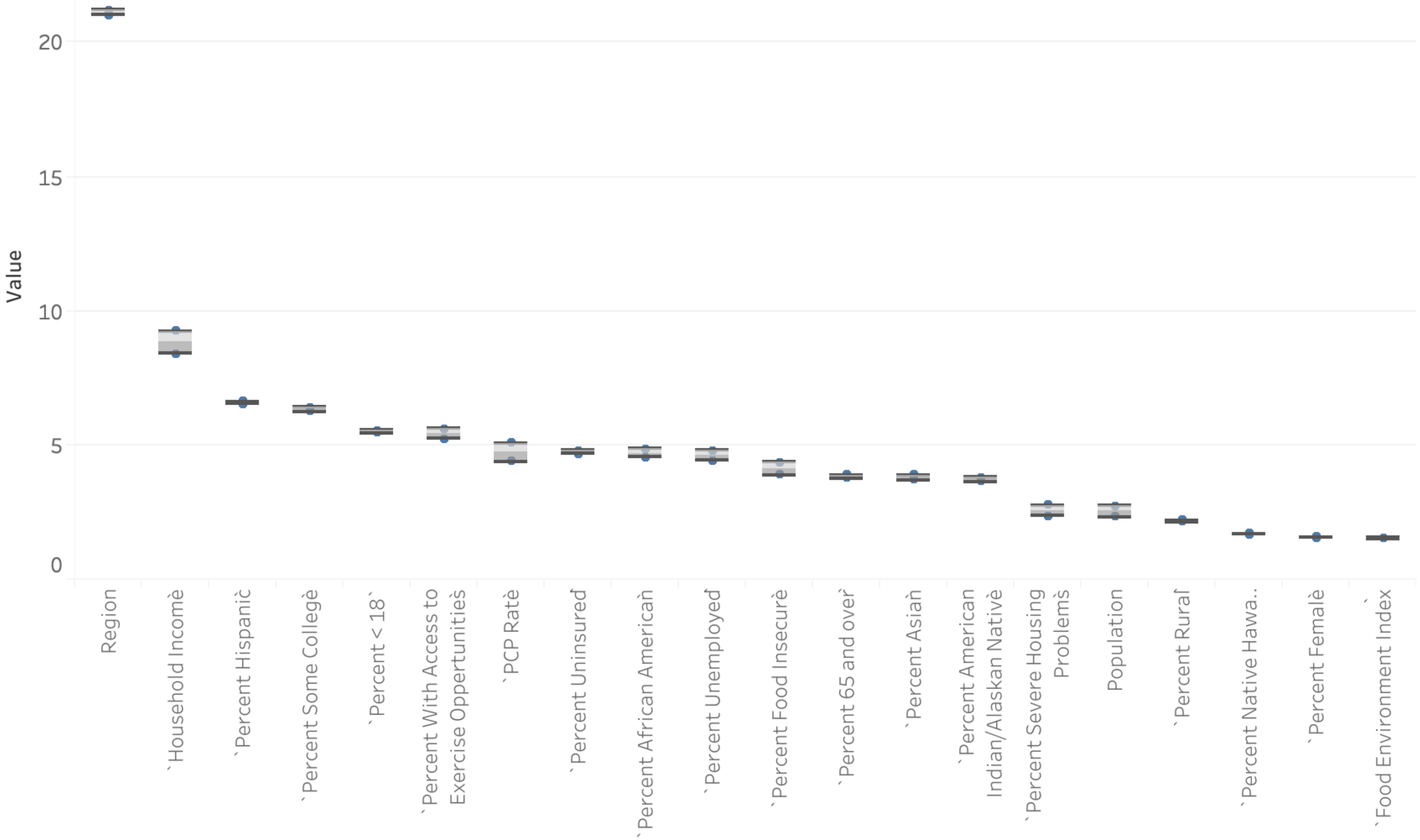

The relative importance of each variable in the GBM model in each of 30-folds of cross validation is shown. The x-axis is labeled with the variables used in the GBM model. The y-axis shows the relative importance of each variable.

© 2019 Scheinker D et al. *JAMA Network Open*.
